# Arteriovenous Loop‐Assisted Free Functional Gracilis Transfer in Vessel‐Compromised Patients: A Salvage Strategy for Facial Reanimation

**DOI:** 10.1002/micr.70238

**Published:** 2026-05-11

**Authors:** Justus Osterloh, Jakob B. W. Weiss, Mark Fricke, Cam Tu Nguyen, Branislav Kollár, Steffen U. Eisenhardt

**Affiliations:** ^1^ Department of Plastic and Hand Surgery, Faculty of Medicine Medical Center‐University of Freiburg Freiburg Germany

**Keywords:** arteriovenous loop, facial reanimation, free functional gracilis transfer, reconstructive microsurgery, vessel‐compromised patients

## Abstract

**Background:**

Free functional gracilis transfer (FFGT) is the gold standard for dynamic facial reanimation in long‐standing facial paralysis and its success depends on the availability of reliable recipient vessels. In patients with compromised vessels due to prior surgery, radiation, or trauma, arteriovenous (AV) loops are a well‐established reconstructive strategy. However, their role in facial reanimation remains scarcely reported.

**Methods:**

We retrospectively analyzed all patients with facial paralysis receiving facial reanimation with FFGT at our center between January 2010 and March 2025. All patients with absent or compromised standard recipient vessels, who underwent facial reanimation using FFGT in combination with an AV loop, were included. A sequential approach with preoperative Doppler examination, intraoperative flow assessment, AV loop construction, and continuous Cook‐Schwartz Doppler monitoring was used before gracilis harvest. Each case was compared with an age‐matched cohort of patients who underwent conventional FFGT without AV loops to contextualize operative and ischemia times. Primary outcomes were technical success and perioperative complications.

**Results:**

Out of 196 patients who underwent facial reanimation, four patients (4/196, 2.0%) with compromised recipient vessels who received AV loop‐assisted FFGT were identified (mean age 57 years (range: 40–65) years). The mean follow‐up time was 19.3 months (13–30 months). No AV‐loop thrombosis, flap loss, hematoma, wound complications, or donor‐site morbidity occurred. Compared with age‐matched controls, operative times were prolonged, whereas ischemia times remained comparable. Functional smile restoration was achieved in two of four patients within 12 months.

**Conclusions:**

AV loop‐assisted FFGT is a technically feasible salvage approach for facial reanimation in patients with compromised recipient vessels, enabling restoration of vascular access and expanding reconstructive options.

**Trial Registration:**

FRKS (Freiburger Reister Klinischer Studien), No. FRK005908

## 1 Introduction

Patients with facial paralysis not only suffer from significant functional impairments such as oral incontinence or loss of emotional facial expression, but also considerable psychosocial burdens (Vargo et al. 2023; Coulson et al. 2004). Dynamic facial reanimation can significantly improve quality of life in this population, creating a strong demand for reliable and effective surgical solutions (Guerreschi and Labbé 2019). Facial reanimation using functional gracilis transfer (FFGT) remains a cornerstone for restoring dynamic facial movement in patients with long‐standing facial paralysis (Jowett and Hadlock 2018; Rozen 2017; Faris et al. 2018). The gracilis muscle is favored for its consistent anatomy, reliable neurovascular pedicle, and minimal donor‐site morbidity (Thiele et al. 2015). However, its successful use depends on the availability of suitable recipient vessels for microvascular anastomosis, which is often compromised in patients with prior surgery, trauma, radiation, or flap failures (Chang et al. 2023).

In these anatomically compromised cases, arteriovenous (AV) loops offer a valuable solution. AV loops are a well‐established microsurgical technique in which a vein graft is interposed between an artery and a vein to create a temporary vascular conduit, thereby bridging the distance to suitable recipient vessels when local options are absent or inadequate. They are most commonly used in limb and head and neck reconstruction, with studies demonstrating high flap survival rates even in complex clinical settings (Pak et al. 2023; Anderson et al. 2021; Cavadas 2008; Meyer et al. 2016).

However, the use of AV loops in facial reanimation surgery remains scarcely described.

We aim to expand the limited evidence on AV loop‐assisted facial reanimation and to highlight its potential as a salvage option in complex reconstructive cases. We present a single‐center series of four patients with severely compromised recipient vessels undergoing AV loop‐assisted FFGT and describe indications, technical workflow, and perioperative outcomes.

## 2 Methods

We conducted a retrospective case series at our Medical Center, evaluating patients who underwent facial reanimation using FFGT with adjunctive AV loop reconstruction. The study period spanned from January 2010 to March 2025. Patients with long‐standing facial paralysis (> 1 year), severely compromised recipient vessels (absent or compromised ipsilateral facial or superficial temporal artery and vein), and FFGT in combination with AV loop were included. This study was designed in accordance with the STROBE (Strengthening the Reporting of Observational Studies in Epidemiology) guidelines. All patients undergoing FFGT underwent comprehensive clinical evaluation and Doppler examination to assess the recipient vessel availability preoperatively. Indications for an AV loop were predefined as (i) absence of suitable facial or superficial temporal recipient vessels on preoperative Doppler examination or (ii) intraoperative failure of these vessels on exposure and flow testing. For contextual analysis, each patient was compared with an age‐matched control group of patients who underwent conventional FFGT without AV loop reconstruction during the same study period. Control patients were selected within ±1 year of age. Operative time and flap ischemia time were recorded for both groups to assess the additional technical impact of AV loop creation. Functional outcomes were evaluated at 12‐month follow‐up, with successful smile reanimation defined as the presence of dynamic commissure excursion.

**FIGURE 1:**
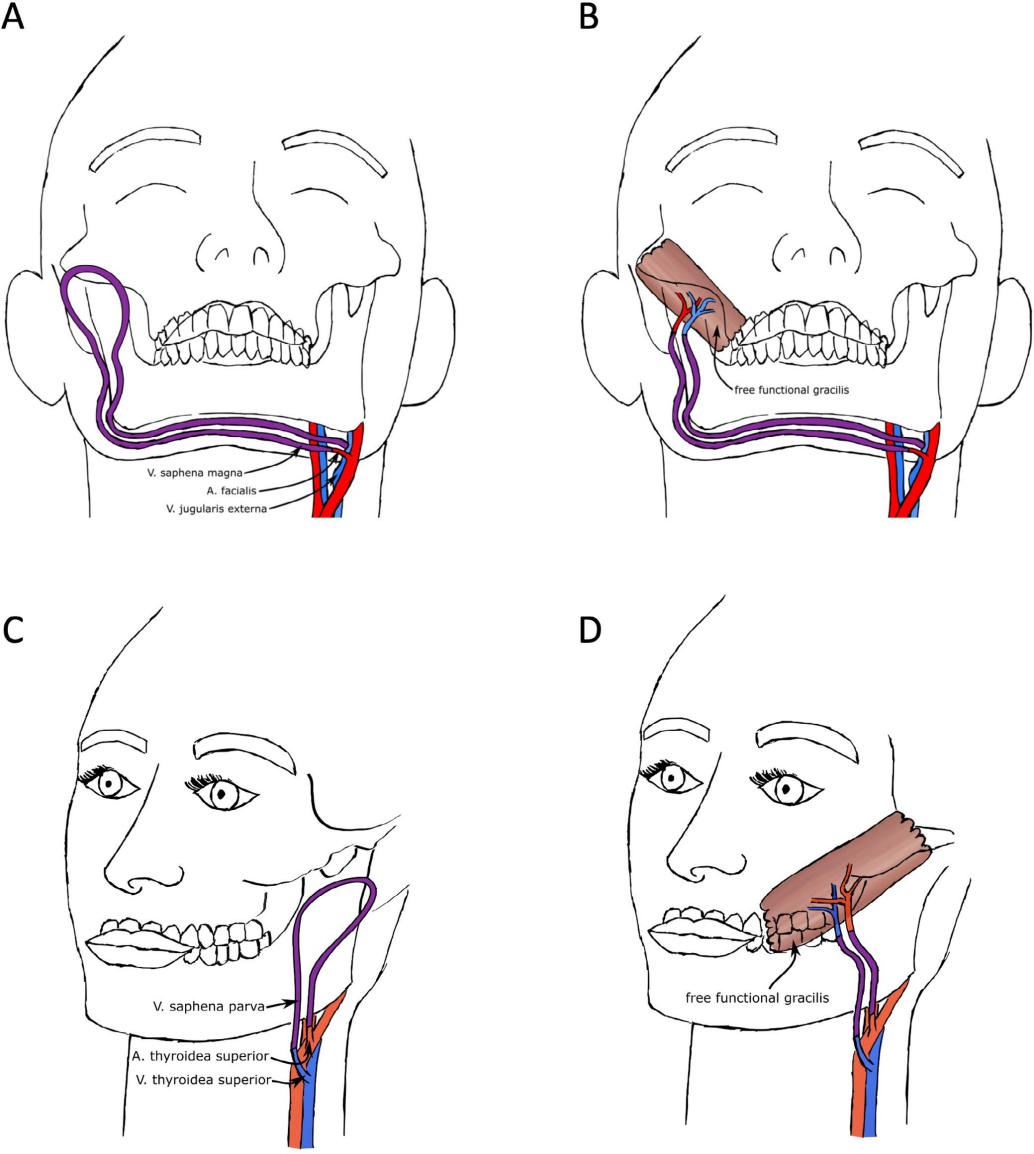
Vascular configurations for AV Loop‐assisted free functional gracilis transfer (FFGT) in vessel‐compromised patients: (A, B) The AV loop was constructed using a vein graft and anastomosed to the contralateral facial artery and jugular vein to provide suitable recipient vessels for FFGT (A). In a second‐staged procedure, the AV loop was divided and anastomosed to the arterial and venous pedicles of the gracilis flap (B). (C, D) The AV loop was created between the superior thyroid artery and jugular vein on the affected side (C). Completion of the single‐stage procedure, with microvascular anastomosis of the gracilis muscle pedicle to the limbs of the AV loop (D).

In cases with uncertain vascular anatomy, a sequential approach was applied. After exposure and intraoperative assessment of potential recipient vessels, a vein graft was harvested, and an AV loop was constructed. AV loops were constructed using vein grafts harvested from the superficial venous system of the lower limb and anastomosed to suitable ipsilateral or contralateral recipient vessels in the face or neck. The different vascular configurations of AV loop‐assisted FFGT used in this series are illustrated in Figure 1. Vein grafts were tailored to achieve a tension‐free course and suitable size match. Grafts were flushed with heparinized saline and marked for orientation.

AV loop and FFGT were performed either as a single‐stage or two‐stage procedure. Single‐stage procedures were preferred when vessel quality and general condition allowed immediate FFGT; two‐stage was chosen to confirm loop patency in complex contralateral routing. The vein graft was first anastomosed to the recipient artery and vein under temporary clamping. After completion of these anastomoses, the clamps were released and blood flow was established through the AV loop. In single‐stage procedures, flow was maintained for approximately 1 h, whereas in the two‐stage procedure, the loop was allowed to mature over 5 days. Intraoperative and postoperative loop and flap monitoring were performed using a Cook‐Schwartz Doppler‐Probe, which has been previously evaluated as a reliable method for perfusion assessment in facial reanimation procedures (Horner et al. 2022). During continuous loop monitoring, the gracilis muscle was harvested in standard fashion with its neurovascular pedicle to minimize the risk of secondary thrombosis and to reduce overall ischemia time. The motor nerve (branch of obturator nerve) was coaptated to the masseteric nerve. Prior to flap anastomosis, the loop was clamped proximally and distally, divided, and the arterial and venous limbs were anastomosed to the corresponding vessels of the flap. Following completion of the flap anastomoses, the arterial clamp was released first, and flap perfusion and venous outflow were assessed before releasing the venous clamp. Vascular anastomoses were performed to the mature AV loop limbs under operating microscope magnification using 8‐0 or 9‐0 nylon sutures as previously described (Thiele et al. 2015; Kiefer et al. 2019, 2018).

**FIGURE 2:**
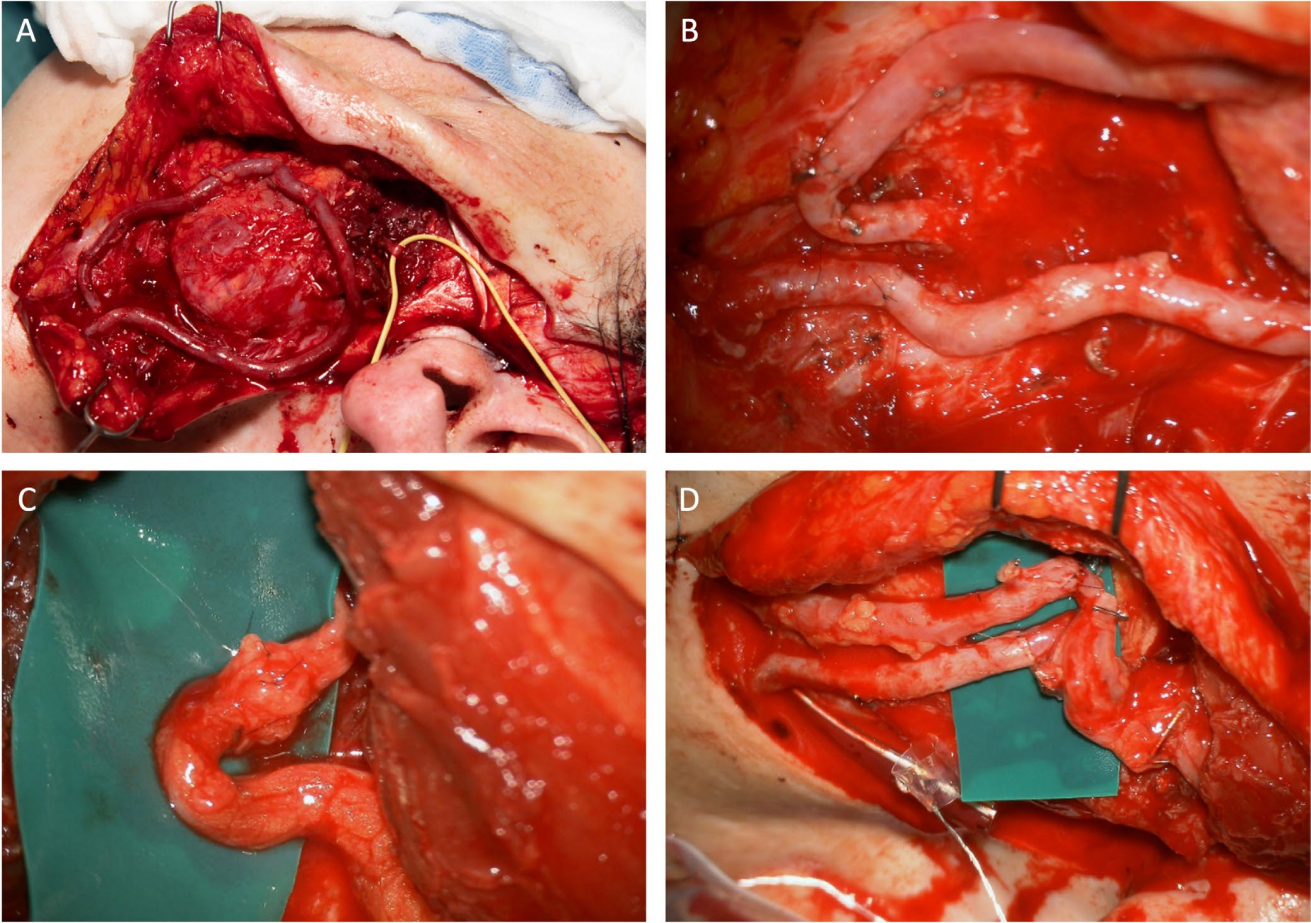
Intraoperative findings during AV loop‐assisted FFGT: (A) Placement of the arteriovenous loop; the masseteric nerve is identified and marked with a yellow vessel loop. (B) Microsurgical view showing arterial and venous anastomoses of the AV loop. (C) Epineural coaptation of the masseteric nerve to the motor branch of the obturator nerve of the gracilis muscle. (D) Anastomosis between the AV loop and the vascular pedicle of the gracilis muscle flap. The FFGT is visible in the lower right corner.

Representative intraoperative steps including AV loop construction, microvascular anastomosis, and nerve coaptation are shown in Figure 2. Postoperative follow‐up included clinical examination and photographic documentation. Primary outcomes were technical success, perioperative complications, and functional outcome after 12 months. During the preparation of this work, the authors used ChatGPT 5.0 (OpenAI) to assist with language editing and formatting suggestions. After using this tool, the authors reviewed and edited the content as needed and take full responsibility for the content of the publication.

## 3 Results

A total of 196 patients with facial paralysis underwent facial reanimation with FFGTs at our center between January 2010 and March 2025. Out of these, four patients (one male, three female; mean age 57 years, range: 40–65 years) with unilateral facial paralysis and severely compromised recipient vessels were treated with FFGT assisted by AV loop reconstruction. The etiology for the facial paralysis was oncological resection of a parotid tumor (parotid carcinoma; *n* = 3) or congenital (*n* = 1). All patients presented with absent or unsuitable recipient vessels for direct microvascular anastomosis on preoperative or intraoperative assessment. AV loops were constructed using great saphenous vein in three of four cases and small saphenous vein in one case, with a mean graft length of 24.75 cm (range: 17–40 cm).

In two patients (Patients 2 and 3), the vein graft was anastomosed to the superior thyroid artery and vein on the ipsilateral side, whereas in Patient 4 the facial vein was intact but distant and the loop was anastomosed to the ipsilateral superior thyroid artery and the facial vein. In these three patients, a temporary AV loop was created, allowing blood flow through the graft for at least 1 h prior to final anastomosis. The loop was subsequently divided, and the arterial and venous limbs were anastomosed to the flap in a single‐stage procedure. In Patient 1, the AV loop was anastomosed to the contralateral facial artery and to a branch of the contralateral internal jugular vein in a two‐stage procedure. The mean total surgery time for AV loop‐assisted FFGT was 393 min (range, 296–481 min), and the mean flap ischemia time was 81 min (range, 70–93 min).

**FIGURE 3:**
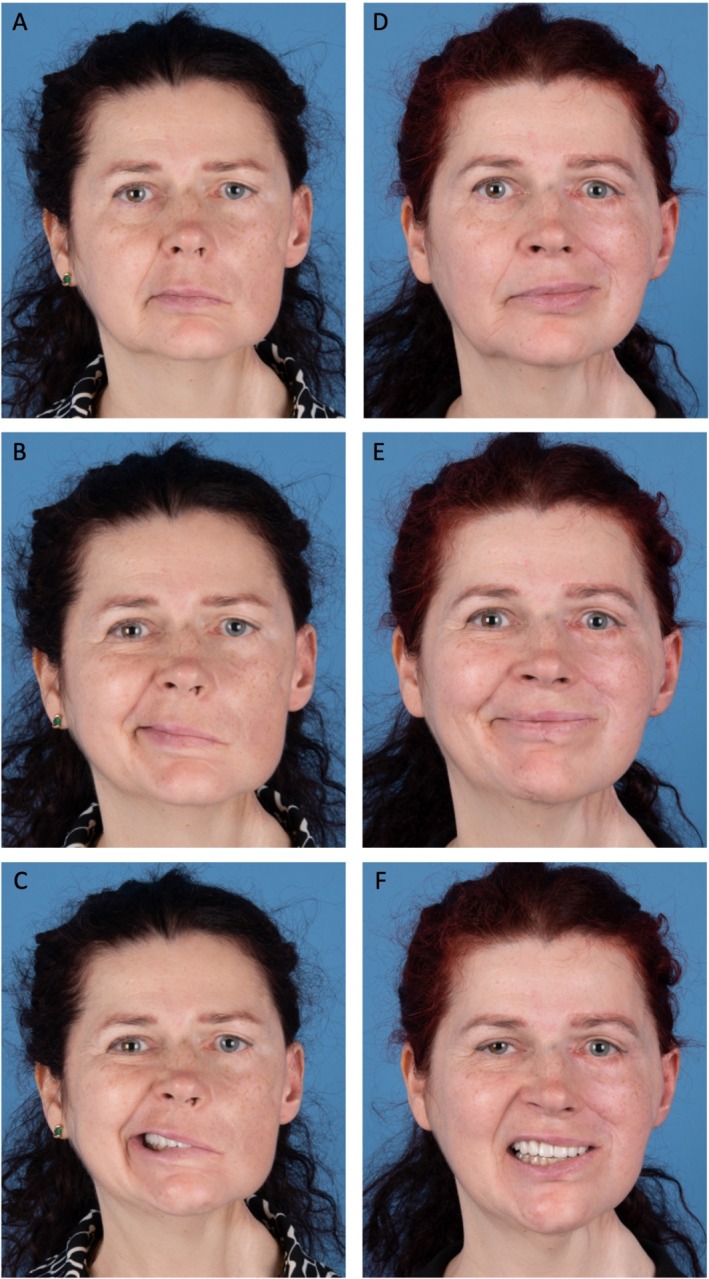
Pre‐ and postoperative facial appearance following AV loop‐assisted free functional gracilis transfer (FFGT): (A–C) Preoperative frontal views of a patient with long‐standing facial paralysis and a vessel‐compromised patient, prior to FFGT with AV loop reconstruction. (A) At rest. (B) Attempted smile without dental show. (C) Attempted smile with dental show. (D–F) Postoperative views at 12 months following FFGT and nerve coaptation to the masseteric nerve. (D) At rest. (E) Voluntary smile without dental show. (F) Voluntary smile with dental show.

To contextualize these values, each patient was compared with an age‐matched cohort (*n* = 6–9 per case) who underwent conventional FFGT without AV loop reconstruction during the same study period. In these comparison groups, the mean operative times ranged from 273 to 332 min, and mean flap ischemia times from 68 to 101 min (Table 2). Thus, the use of an AV loop increased the operative time by approximately 60–150 min, while flap ischemia times remained within a comparable range. All patients received a FFGT with coaptation of the obturator nerve branch of the gracilis muscle to the masseteric nerve. Microvascular anastomosis was performed between the vascular flap pedicle and the arterial and venous limbs of the AV loop. The mean flap length was 12.88 cm (range: 12.5–13.0 cm) and the mean weight was 32.5 g (range: 26–39 g). No AV loop thrombosis or flap loss occurred. No hematoma, wound dehiscence, infections, or donor site complications were observed. Representative pre‐ and postoperative clinical photographs demonstrating facial appearance at rest and during smiling are shown in Figure 3. Patient characteristics, operative details, and outcomes are summarized in Table 1. The mean follow‐up time was 19.3 months (range: 13–30 months). At 12‐month follow‐up, successful dynamic smile reanimation was achieved in 2 of 4 patients (Patient 1 and 2; 50%). Among age‐matched control patients undergoing conventional FFGT without AV loop (*n* = 29), successful smile reanimation was achieved in 24 of 26 patients (92.3%) with available 12‐month follow‐up. Two patients (7.7%) did not achieve functional reanimation, and three patients were lost to follow‐up before reaching 12 months.

**TABLE 1:** Clinical summary of patients who underwent AV loop‐assisted free functional gracilis transfer (FFGT), including patient characteristics, etiology of facial paralysis, previous facial paralysis associated surgeries, reason for vessel depletion, laterality of AV loop, donor vein, coaptated nerve, flap characteristics and commissure excursion.

Patient	Age	Gender	Etiology of facial paralysis	Previous facial paralysis asscoiated surgeries	Reason for vessel depletion	Laterality of AV loop	Recipient vessels	Donor vein length (in cm)	Coaptated nerve	Flap weight (in g)	Flap length (in cm)	Successful smile restauration
1	60	Female	Parotid carcinoma	− Static oral commissure suspension − Two revisions of the oral commissure suspension − Two exchanges of the upper eyelid platinum weight − Two brow lifts	Soft tissue coverage using an anterolateral thigh (ALT) flap after neck dissection	Contralateral	Facial artery and jugular vein	Great saphenous vein (40)	Masseteric nerve	35	12.5	Yes
2	53	Female	Parotid carcinoma	− McLaughlin oral commissure suspension − Upper eyelid gold weight implantation − Soft tissue augmentation with Tutopatch (Tutogen Medical GmbH, Neunkirchen am Brand, Germany)	Neck dissection and adjuvant radiotherapy	Ipsilateral	Superior thyroid artery and vein	Small saphenous vein (17)	Masseteric nerve	26	13	YES
3	40	Female	Congenital	− Gilles‐Transfer − Cross‐face nerve graft and FFGT attempt − McLaughlin suspension − SMAS facelift − Lip vermilion correction	FFGT attempt	Ipsilateral	Superior thyroid artery and vein	Great saphenous vein (24)	Masseteric nerve	30	13	No
4	65	Male	Parotid carcinoma	− Cervical plexus interposition grafting − Upper eyelid platinum weight implantation − Tarsal tongue flap − Lateral canthopexy	Neck dissection and adjuvant radiotherapy	Ipsilateral	Superior thyroid artery and facial vein	Great saphenous vein (18)	Masseteric nerve	39	13	No

## 4 Discussion

In this study, we report four patients with facial paralysis and compromised recipient vessels who received facial reanimation with FFGT and AV loops. All flaps survived without AV loop thrombosis, indicating that AV loops represent a feasible strategy for salvage reconstruction in head and neck surgery. All flaps were monitored for 1 week postoperatively using a Cook‐Schwartz Doppler‐probe. This aligns with the current literature with larger cohorts in head and neck reconstruction, where survival rates of AV loop‐assisted free flaps are described in a range between 85% and 100% (Pak et al. 2023; Anderson et al. 2021; Meyer et al. 2016).

The patient cohort presented with heterogeneous etiologies and extensive prior interventions. All patients had undergone multiple procedures at external institutions before presentation, reflecting the reconstructive complexity and need for individualized surgical planning.

One patient (Patient 1) underwent a two‐stage procedure with a contralateral AV loop anastomosed to the facial artery and jugular vein, whereas the remaining three patients underwent single‐stage procedures with AV loops anastomosed to ipsilateral branches of the superior thyroid artery and either the superior thyroid or facial vein. The decision between a single‐ versus two‐stage AV loop procedure remains a topic of debate in complex reconstructive surgery. While single‐stage AV loop reconstruction offers the advantages of reduced overall treatment time, it entails longer initial surgery and less opportunity to assess loop patency before free flap transplantation. Two‐stage approaches allow confirmation of loop patency and shorter individual procedures, which may benefit high‐risk or medically fragile patients, but require a second operation. In contemporary practice, the choice between interposition vein grafts and AV loop‐based reconstruction, as well as between single‐stage and two‐stage approaches, remains a topic of discussion. However, single‐stage procedures are increasingly favored, emphasizing the importance of careful preoperative planning considering the patient comorbidities, defect complexity and intraoperative findings (Pak et al. 2023; Knackstedt et al. 2018).

Compared to age‐matched controls undergoing conventional FFGT without AV loops, surgery times were prolonged by approximately 1–2 h, reflecting the additional vascular dissection, loop creation, and sequential intraoperative flow assessment. Nevertheless, ischemia times remained within a comparable range, indicating that the AV loop procedure did not compromise flap perfusion or anastomotic workflow (Table 2). The sequential approach, which included continuous Doppler monitoring before gracilis harvest, provided increased intraoperative safety under uncertain vascular conditions.

**TABLE 2:** Surgery and ischemia times for AV loop‐assisted FFGT compared with age‐matched controls without AV loop reconstruction: Operative time and flap ischemia time for each patient undergoing AV loop‐assisted free functional gracilis transfer (FFGT) compared with age‐matched control groups who received conventional FFGT without AV loop reconstruction. Values are presented as mean (range). The AV loop group showed longer overall operative times due to additional vascular preparation, while ischemia times remained comparable between groups.

Patient	Surgery time (min)	Ischemia time (min)	Comparison cohort (*n*)	Mean surgery time (min) (range)	Mean ischemia time (range)
1	481	93	6	332 (238–350)	101 (76–140)
2	400	71	6	273 (218–308)	87 (73–96)
3	393	88	8	288 (216–362)	85 (72–108)
4	296	70	9	280 (230–397)	68 (82–112)

Although the AV loops reliably restored vascular circulation for flap survival in all presented cases, functional muscle activation involves distinct challenges, and vascular success does not guarantee sufficient neuromuscular function. For functional reanimation, the masseteric nerve‐innervated FFGT is a highly effective and safe option with robust and rapid smile excursion (Weiss et al. 2023; Roy et al. 2019; Fernández‐Carrera González et al. 2023), as also reflected in the age‐matched control cohort undergoing conventional FFGT without AV loop reconstruction. In the present cohort, successful dynamic reanimation was achieved in only 2 of 4 patients despite technically successful flap transfer and adequate vascular perfusion, as confirmed by continuous postoperative monitoring.

Several potential factors may explain the variability in neuromuscular recovery and functional outcome despite vascular patency. All patients in this series presented with complex oncologic backgrounds, including prior surgery and/or radiotherapy, which may impair the local neural environment and regenerative capacity. Patient 4 developed recurrent disease of parotid carcinoma after reanimation surgery. Tumor infiltration of the neurovascular pedicle might negatively impact nerve growth and could be an explanation for the absence of movement of the functional flap in Patient 4. Other factors might include impaired neuroplasticity or induced fibrosis due to radiation therapy in oncologic patients. Patient 3 had previously undergone unsuccessful dynamic facial reanimation procedures. These prior interventions likely contributed to extensive scarring, distorted anatomy, and compromised nerve function. In a recent study, Kollàr et al. evaluated secondary FFGT following failed primary dynamic reanimation and found that all patients achieved smile restoration, although those with prior static interventions demonstrated greater excursion and symmetry than those with previous dynamic procedures (Kollar et al. 2023). In contrast, Patient 3 in our cohort did not regain visible muscle contraction or functional smile, despite technically successful flap transfer and nerve coaptation. The findings suggest that the underlying disease burden and local tissue environment, rather than the vascular reconstruction itself, may represent the primary limiting factors. The exact causes of functional failure cannot be determined with certainty.

Additionally, these findings have important implications for patient selection and preoperative counseling. All patients underwent thorough preoperative clinical evaluation and expressed a strong desire for surgical treatment. They were informed in detail about the high risks and uncertain functional outcomes, as reflected by the 50% success rate of this cohort. The risk may be considered unacceptably high by some patients and must therefore be clearly communicated. Careful patient selection and realistic counseling are therefore crucial. The choice of treatment is critical in this complex patient population, and the observed failure rate highlights the need to consider alternative reconstructive strategies. Reports on alternative approaches for free functional muscle transfers in patients with compromised recipient vessels remain limited. De Cicco et al. recently described an inverted gracilis flap with intraoral anastomosis to the contralateral side in a patient with compromised recipient vessels, underscoring the technical creativity required in such cases (De Cicco et al. 2024). However, no signs of contraction of the FFGT could be detected in the follow‐up examinations after local recurrence and distant metastasis of the parotid carcinoma occurred.

The use of the free latissimus dorsi (LD) muscle flap for facial reanimation in patients with facial paralysis has also been described as an alternative (Takushima et al. 2013; Okazaki et al. 2024). LD muscle flap offers a reliable anatomy with a long vascular pedicle potentially enabling reconstruction without an AV loop even in the setting of vessel‐compromised patients. However, compared with the gracilis muscle, the LD flap provides less excursion, which may limit oral commissure movement and thus functional outcomes (Takushima et al. 2013). Ibarra et al. reported a case series of 6 patients who underwent FFGT with interposition of vein grafts (*n* = 3) and use of the vascular pedicle from previous flaps (Ibarra and Lasso 2022).

Alternative local approaches, including regional muscle transfers such as temporalis transfer (Namavarian et al. 2026) or masseteric muscle transfer (Lesavoy et al. 2014), may offer shorter operating times and more predictable functional outcomes in patients with long‐standing facial paralysis, particularly when the local tissue and neural environment are significantly compromised. However, all these techniques differ in excursion, spontaneity, and vector of movement and may not be suitable or acceptable for all patients (Oyer et al. 2018; Jones et al. 2024; Garcia et al. 2015). Therefore, treatment decisions should be individualized, considering the reconstructive environment, patient expectations, and the potential risks and benefits of each approach.

Major limitations of this study are the small sample size and its retrospective design which limit the broader generalizability of the findings. Nonetheless, the study adds to the limited body of evidence in this challenging subset of patients and may help guide surgical decision‐making in cases with compromised recipient vessels. To our knowledge, this represents the first reported case series of AV loop‐assisted FFGT for facial reanimation.

## 5 Conclusion

AV loop‐assisted FFGT is a feasible option to establish vascular access in patients with facial paralysis and severely compromised recipient vessels. It should be considered in patients with maximal therapeutic desire after thorough preoperative counseling.

## Funding

The authors have nothing to report.

## Ethics Statement

This study was approved by the Ethics Committee of the Albert‐Ludwig‐University Freiburg, Germany (Approval No. 25‐1200‐S1‐retro).

## Consent

Written informed consent was obtained from all patients for publication of identifiable images.

## Conflicts of Interest

The authors declare no conflicts of interest.

## Data Availability

The data that support the findings of this study are available from the corresponding author upon reasonable request.
